# Prevalence of obstructive lung disease in an African country using definitions from different international guidelines: a community based cross-sectional survey

**DOI:** 10.1186/s13104-015-1731-6

**Published:** 2016-02-25

**Authors:** Eric Walter Pefura-Yone, André Pascal Kengne, Adamou Dodo Balkissou, Christiane Gaelle Magne-Fotso, Martine Ngo-Yonga, Julie Raïcha Boulleys-Nana, Nelly Rachel Efe-de-Melingui, Patricia Ingrid Ndjeutcheu-Moualeu, Charles Lebon Mbele-Onana, Elvira Christelle Kenmegne-Noumsi, Barbara Linda Kolontchang-Yomi, Boris Judicaël Theubo-Kamgang, Emilienne Régine Ebouki, Chrystelle Karen Djuikam-Kamga, Francine Amougou, Liliane Mboumtou, Elsie Linda Petchou-Talla, Christopher Kuaban, Respiratory Health Survey Group in Cameroon (RHSGC)

**Affiliations:** Department of Internal Medicine and Subspecialties, Faculty of Medicine and Biomedical Sciences, University of Yaounde I, Yaounde, Cameroon; Pneumology service, Yaounde Jamot Hospital, P.O Box: 4021, Yaounde, Cameroon; South African Medical Research Council & University of Cape Town, Cape Town, South Africa; Institut Supérieur de Technologie Médicale, Yaounde, Cameroon; Faculty of Heath Sciences, University of Bamenda, Bamenda, Cameroon

**Keywords:** COPD, Air flow limitation, Associated factors, Africa

## Abstract

**Background:**

Obstructive lung disease (OLD), a major global public health problem, has been less investigated in African countries. We assessed the prevalence and determinants of OLD in Yaounde (the capital city of Cameroon), using internationally agreed definitions.

**Methods:**

Participants were adults (age >19 years) screened during a community-based survey between December 2013 and April 2014. Air flow limitation (AFL) was based on a pre-bronchodilator forced expiratory volume in 1 s (FEV1) and forced vital capacity (FVC) below the lower limit of normal (LLN, AFL–LLN). Chronic obstructive pulmonary disease (COPD) was based on post-bronchodilator FEV1/FVC ratio < LLN (COPD–LLN).

**Results:**

Of the 1287 subjects included, 51.9 % were female, 9.3 % were current smokers and their mean age was 34.4 ± 12.8 years. Forty-nine (3.8 %, 95 % CI 2.8–4.9 %) participants had AFL–LLN. Thirty-one subjects had COPD–LLN; giving a prevalence of COPD–LLN of (2.4 %, 95 % CI 1.6–3.3 %). In multivariable analysis, male gender (AOR 2.42; 95 % CI 1.12–5.20) and lifetime wheezing (AOR 2.88; 95 % CI 1.06–7.81) were the determinants of COPD-LLN. Otherwise, male sex (AOR 1.93, 95 % CI 1.00–3.73), age 40–59 years (AOR 1.99, 95 % CI 1.04–3.81) and lifetime wheezing (AOR 2.65, 95 % CI 1.13–6.20) remained as independent determinants of AFL–LLN.

**Conclusions:**

Obstructive lung disease based on more accurate definitions was relatively infrequent in this population. It is important to sensitize the medical staff and the general public about this condition which should be actively investigated in individuals aged 40 years and above.

## Background

Chronic obstructive pulmonary disease (COPD) is a major global public health problem. The World Health Organization (WHO) has estimated that about 65 million people suffer from moderate to severe COPD worldwide [1]. A striking issue with COPD is that the condition is often underestimated by the patient, and largely under diagnosed and as a consequence undertreated by medical doctors [2]. The prevalence of COPD varies across regions and can be as high as 10 % or beyond in regions with high prevalence of smoking [3]. COPD is currently the fourth leading cause of death globally, and is set according to WHO estimates to become the third leading cause of death and the fifth provider of disability worldwide by 2030 [1].

Smoking is the leading risk factor for COPD worldwide with the attributable risk fraction of COPD from active smoking ranging from 40 to 70 % across countries [4–6].With an estimated prevalence of smoking ranging from 8 to 43 % in men and 5 to 30 % in women, and the expanding tobacco industry across the continent, COPD is set to become a major health challenge in African countries, alongside other communicable and non-communicable diseases [7]. Although smoking is a major risk factor for COPD, it remains that a large number of those with COPD have no history of smoking habits [8, 9]. Other contributing factors to the global burden of COPD include age greater than 40 years, air pollution, exposure to biomass, exposure to certain gases, low socio-economic status, genetic factors, history of pulmonary tuberculosis and HIV infection [4, 8].

Published studies on COPD largely originate from developed countries. Very few epidemiological studies have been conducted on COPD in sub-Saharan Africa, although COPD represents the fourth cause of death in low and middle income countries [10]. Indeed, according to WHO’s estimates COPD accounted for 116,000 deaths in Africa in 2001, a contribution similar to that observed in Europe [11]. In a recent systematic review, Finney et al. [6] found only nine cross-sectional studies of acceptable methodological quality on COPD in the general population in Africa. Efforts to close this knowledge gap will have to overcome the challenge of varying definitions of air flow obstruction and COPD across recommendations. For instance, American Thoracic Society/European Respiratory Society (ATS/ERS) recommends that the forced expiratory volume in 1 s/forced vital capacity (FEV1/FVC) ratio < lower limit of normal (LLN) should be used to define COPD, while the global initiative for obstructive lung disease (GOLD) rather recommends that the fixed ratio of FEV1/FCV (<0.70) should be used [12, 13]. In all instances, demonstrating a chronic airflow obstruction not fully reversible after bronchodilator inhalation is essential to define COPD [3]. It is against this background that the current study was undertaken with a dual objective of: 1) determining the prevalence of obstructive lung disease (OLD) including air flow limitation (AFL) and COPD in a major city in the Central Africa Region according to the different internationally recommended definitions; and 2) investigating the determinants of these conditions among adults in this setting.

## Methods

### Type of study, study setting and population

This was a community-based cross-sectional survey conducted between December 2013 and April 2014 (5 months duration) across all the seven districts of Yaounde, the Capital City of Cameroon with about 2 million inhabitants including 1.4 million adults [14]. Consenting adults aged 19 years and above were considered for inclusion in the study [15]. They had to be free of any of the following conditions: pneumonia in the last 4 weeks, active thoracic tuberculosis, physical or mental impairment affecting the ability to perform spirometry. The study was approved by the institutional ethic review committee of the Faculty of Medicine and Pharmaceutical Sciences of the Douala University and the administrative authorities of the Health Delegation for the Centre Region.

### Sampling

A three level stratified cluster sampling was applied. At the first level, 16 enumeration areas (EA, 2–3 per district) were selected using a simple random sampling. EA demarcations were those used in the third general population census conducted in Cameroon in 2005 [14]. Each EA comprised about 140–220 households. At the second level, one in two household was selected using a systematic sampling. The first selected household and the itinerary were those used during the national vaccination campaigns. At the third level, all individuals aged 19 years and above in selected households formed the primary statistical unit for data collection.

### Data collection

Data were collected by final year undergraduate medical students who were specifically trained for this purpose. The survey involved fourteen undergraduate final year medical students and six spirometry technicians. Data collection during face-to-face interviews used a pre-tested questionnaire, derived from questionnaires used in international surveys [16, 17]. Data were collected on: 1) socio-demographic characteristics including age, sex, level of formal education (none, primary, secondary, university), 2) smoking history with participants distinguished as non-smokers (including never-smokers and lifetime smoking below 20 packs in their lifetime), smokers (including those currently smoking and with a lifetime cigarette smoking higher than 20 packs in their lifetime), and former-smokers (including those who had stopped smoking for over 6 months) [16]; 3) past history of respiratory diseases including tuberculosis, asthma, chronic bronchitis; 4) chronic respiratory symptoms including chronic cough and expectoration (lasting for at least 3 months per year), dyspnea which was graded in four stages (stage 1—shortness of breath while climbing at normal pace on mild inclined surface or a floor of the building, stage 2—shortness of breath while walking at normal pace with age mates on a flat surface, stage 3—shortness of breath while walking at own’s pace on a flat surface, stage 4—shortness of breath at rest or any small effort); 5) exposure to biomass based on the exposure to cooking smoke from solid fuel, and was considered as exposed all participants living for at least 6 months in a household where solid fuel was used for cooking purposes; 6) anthropometric measurements including height (meter) measured to the nearest centimeter using a stadiometer, weight (kg) measured with a CAMRY scale (CAMRY, Guangzhou, China), and body mass index calculated as weight (kg)/[height (m)*height (m)].

### Spirometric measures

Spirometric data were obtained for all eligible participants as per standard methods [18], using turbine pneumotachograph (Spiro USB,Care fusion, Yorba Linda-USA) or a Fleisch pneumotachograph (Spirolyser SPL-10 USB, FIM-Medical, Lyon-France), meeting the ATS 1994 standards. All measurements were performed after at least 15 min rest, with the participant in a seated position, with the back straight, and the nose clipped to allow air flow only by mouth. The ATS/ERS acceptability and reproducibility criteria were applied [19]. At least three tests were done by each participant to establish the FVC curve. Spirometric variables measured included: FEV1, FVC and the FEV1/FVC ratio. FEV1 and FVC values retained were the best out of the three tests which fulfilled the acceptability criteria (maximal difference below 5 % or 150 ml). All participants with a FEV1/FCV below the LLN or 0.70 received 400 µg of inhaled salbutamol and had the tests repeated 15 min later to assess the reversibility of AFL. Predicted values were estimated using the reference spirometric values for an African population derived by Musafiri et al. [20]. Quality control was done by regularly supervising investigators and technicians who performed spirometry. Spirometry curves were reviewed weekly by one of chest physician involved in the study (EWPY and ADB) and feedback was made to technicians.

All spirometric tests results were reviewed by an experienced chest physician (EWPY or ADB).

### Operational definitions

Operational definitions were those recommended by the ATS/ERS (the use of LLN of FEV1/FVC ratio) [13] or by the global initiative for chronic obstructive lung disease (GOLD) [fixed cut-off of 0.70 for the FEV1/FVC ratio] [21]. Air flow limitation (AFL) was defined by a pre-bronchodilator FEV1/FVC < LLN (AFL–LLN) or below 0.70 (AFL–fixed cut-off). COPD was defined by a post-bronchodilator FEV1/FVC below the LLN (COPD–LLN) or below 0.70 (COPD–fixed cut-off). The severity of AFL or COPD was based on the modified 2006 GOLD stages [13]: stage I (mild)—FEV1 >80 % of the predicted value; Stage II (moderate)—FEV1 comprised between 50–80 % of the predicted value; Stage III and IV (severe)—FEV1 <50 % of the predicted value.

### Statistical methods

The sample size was calculated by estimating the population of adults aged 19 years and above to be 1.4 million. For a type I error of 5 %, an estimated prevalence of 4.5 % for COPD [22], and a precision of 1.5 %, the required sample size was 733 individuals. Considering a correction factor of 1.5 for the cluster effect, and a non-response rate of 10 %, the final estimated sample size was 1210 individuals. Data analysis used the IBM-SPSS v.20 for Windows (IBM, Chicago, USA). Categorical variables were presented as count and frequencies, and continuous variables as mean and standard deviation (SD) or median and 25th–75th percentiles. Group comparisons used Chi square test and Fisher exact test for qualitative variables, and Student’s t test and equivalents for quantitative variables. Logistic regression models were used to investigate the determinants of AFL and COPD. Significant variables in univariable analysis (based on a threshold p <0.10) were entered together in the same multivariable model and the significant ones retained as the final determinants. A p value <0.05 was used to characterize statistically significant results.

## Results

### Study population

**Fig. 1:**
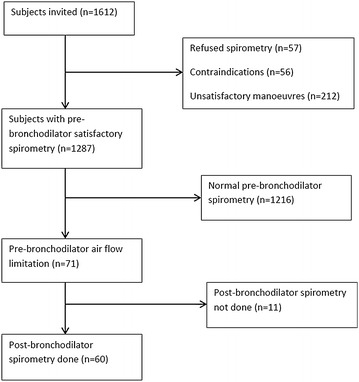
Flow chart for the derivation of the analytic sample

A total of 1612 participants were invited to take part in the study, of whom 57 declined, and a further 56 participants were excluded for a contraindication to spirometry or incomplete questionnaires. Of the 1499 participants who had spirometric tests done, 212 had incorrect maneuvers. Therefore 1287 were included in the final analytic sample even though, 11 participants among those with a pre-bronchodilator AFL did not have a post-bronchodilator spirometric test (Fig. 1).

The baseline characteristics of participants are depicted in Table 1. Of the 1287 participants included, 668 (51.9 %) were women and 619 (48.1 %) were men. The mean age was 34.4 ± 12.8 years. Of the 1283 participants with known status for smoking, 9.3 % were smokers and 6.8 % were ex-smokers. There were more male than female smokers (17 vs 2.3 %, p <0.001). The median (25th–75th percentile) quantity of tobacco smoked was 4.3 (1.2–13.7) pack-years. Only 28 (15.6 %) smokers and ex-smokers had a cumulative quantity of smoked of 20 pack-years or more. Dyspnea was more frequent in women (6.1 %) than in men (2.7 %), p = 0.003. A history of pulmonary tuberculosis was found in 21 (1.6 %) participants. Chronic expectoration and lifetime wheezing were found in 0.8 and 6.6 % of the participants, respectively.

**Table 1:** Characteristics of subjects included in the study in Yaounde, Cameroun

Characteristics	Overall n = 1287 (%)	Men n = 619 (%)	Women n = 668 (%)	p
Age, years				
Mean (SD)	34.4 (12.8)	34.2 (13.1)	34.5 (12.4)	0.309
Median (25th–75th percentiles)	30 (24–42)	30 (24–42)	31 (25–42)	0.312
Min–Max	19–83	19–81	19–83	/
Age group, years				
19–39	911 (70.8)	446 (72.1)	465 (69.6)	0.124
40–59	306 (23.8)	134 (21.6)	172 (25.7)	
≥60	70 (5.4)	39 (6.3)	31 (4.6)	
Level of education				
≤secondary	854/1285 (66.5)	363/618 (58.7)	491/667 (73.6)	<0.001
Higher education	431/1285 (33.5)	255/618 (41.3)	176/667 (26.4)	
Subject/chamber				
≤2	711/1281 (55.5)	384/617 (62.2)	327/664 (49.2)	<0.001
>2	570:1281 (44.5)	233:617 (37.8)	337/664 (50.8)	
Tobacco smoking				
Current smoker	120/1283 (9.4)	105/618 (17)	15/665 (2.3)	<0.001
Ex-smokers	87/1283 (6.8)	76/618 (12.3)	11/665 (1.7)	
Non smokers	10/1283 (83.9)	437/618 (70.7)	639/665 (96.1)	
Cooking fuel				
Clean	557/1252 (44.5)	313/659 (52.8)	244/593 (37)	<0.001
Biomass	99/1252 (7.9)	3/659 (6.6)	60/593 (9.1)	
Mixed	596/1252 (47.6)	241/659 (40.6)	355/593 (53.9)	
Past history of TB				
No	1266 (98.4)	608 (98.2)	658 (98.5)	0.692
Yes	21 (1.6)	11 (1.8)	10 (1.5)	
Past history of pneumonia				
No	1250/1286 (97.2)	598/619 (48.1)	652/667 (97.8)	0.214
Yes	36/1286 (2.8)	21/619 (3.4)	15/667 (2.2)	
HIV infection				
No	1005 (78.1)	456 (73.7)	549 (82.2)	<0.001
Yes	11 (0.9)	4 (0.6)	7 (1)	
Don’t know	271 (21.1)	159 (25.7)	112 (16.8)	
Chronic cough				
No	1262 (98.1)	607 (98.1)	655 (98.1)	0.992
Yes	25 (1.9)	12 (1.9)	13 (1.9)	
Chronic expectoration				
No	1277 (99.2)	614 (99.2)	663 (99.3)	>0.999
Yes	10 (0.8)	5 (0.8)	5 (0.7)	
Chronic dyspnea				
No	1229 (95.5)	602 (97.3)	627 (93.9)	0.003
Yes	58 (4.5)	17 (2.7)	41 (6.1)	
Wheezes				
No	1198/1283 (93.4)	583/617 (48.1)	615/666 (92.3)	0.122
Yes	85/1283 (6.6)	34/617 (5.5)	51/666 (7.7)	
BMI, kg, mean(SD)	26.4 (5.4)	25.3 (4.3)	27.5 (6.1)	<0.001
AFL–pre-LLN				
Yes	49 (3.8)	32 (5.2)	17 (2.5)	0.014
No	1238 (96.2)	587 (94.8)	651 (97.5)	
AFL–fixed ratio				
Yes	9 (0.7)	6 (1)	3 (0.4)	0.263
No	1278 (99.3)	613 (99)	665 (99.6)	
COPD–LLN				
Yes	31/1276 (2.4)	21/608 (3.5)	10/668 (1.5)	0.023
No	1245/1276 (97.6)	587/608 (96.5)	587/668 (96.5)	
COPD–fixed-ratio				
Yes	7/1276 (0.5)	4 (0.7)	3 (0.4)	0.715
No	1269/1276 (99.5)	604/608 (99.3)	665/668 (99.6)	

*SD* standard deviation, *HIV* human immunodefiency virus, *BMI* body mass index, *AFL* airflow limitation, *LLN* lower limit of normal, *COPD* chronic obstructive pulmonary disease

### Prevalence and severity of obstructive lung disease

The prevalence of AFL and COPD are shown in Table 1. Forty-nine (3.8 %, 95 % CI 2.8–4.9 %) participants had AFL–LLN while 9(0.7 %, 95 % CI 0.2–1.2 %) had AFL–fixed ratio. Thirty-three (69.4 %), 13 (26.5 %) and 2 (4.1 %) participants were respectively classified as having mild, moderate and severe AFL–LLN.

Thirty-one participants had COPD–LLN, giving a prevalence of COPD–LLN of 2.4 % (95 % CI 1.6–3.3 %). The prevalence of COPD–fixed ratio was 0.5 % (95 % CI 0.1–0.9 %). Mild, moderate and severe COPD–LLN was respectively found in 67.7, 19.4 and 12.9 % of participants with COPD–LLN.

### Determinants of air flow limitation

Univariable analysis of determinants of air flow limitation (AFL–LLN) is found in Tables 2. In univariable analyses, men were more affected than women (5.2 vs. 2.5 %, p = 0.014). The prevalence of AFL–LLN was much higher in participants aged 40–59 years (5.6 %), and 60 years and above (7.1 %) than those aged 19–39 years (3 %). Ten (20.4 %) participants with AFL–LLN were current smokers vs. 110 (8.9 %) in those without AFL–LLN (p = 0.008). The prevalence of AFL–LLN was similar in participants exposed to biomass than in the non-exposed (3.7 vs. 3.9 %, p = 0.893). The prevalence of AFL–LLN was higher in participants with self-reported positive status for HIV than in those with unknown HIV status. Lifetime wheezing was more frequent in participants with AFL–LLN than in those without (14.3 vs. 6.3 %, p = 0.0038). In multivariable analysis including all variables with a p value <0.10, male sex (AOR 1.93, 95 % CI 1.00–3.73), age 40–59 years (AOR 1.99, 95 % CI 1.04–3.81) and lifetime wheezing (AOR 2.65, 95 % CI 1.13–6.20) remained as independent determinants of AFL–LNN (Table 3).

**Table 2:** Factors associated to air flow limitation (AFL–LLN) in univariable analysis

Characteristics	AFL–LLN n = 49 (%)	No AFL n = 1238 (%)	COR (95 % CI)	p value
Sex				
Male	32 (65.3)	587 (47.4)	2.09 (1.15–3.80)	0.014
Female	17 (34.7)	651 (52.6)	1	
Age, years, m (SD)	39.1 (15.2)	34.2 (12.6)	1.03 (1.01–1.05)	0.008
Age group				
19–39	27 (55.1)	884 (71.4)	1	
40–60	17 (34.7)	289 (23.3)	1.93 (1.03–3.58)	0.036
≥60	5 (10.2)	65 (5.3)	2.52 (0.94–6.76)	0.071
Level of education				
≤Secondary	32/48 (66.7)	822/1237 (66.5)	1.01 (0.55–1.86)	0.975
Higher education	16/48 (33.3)	415/1237 (33.5)	1	
Subject/chamber				
≤2	26 (53.1)	689/1236 (55.7)	1	0.711
>2	23 (46.9)	547/1236 (44.3)	1.11 (0.63–1.98)	
Tobacco smoking				
Current smoker	10 (20.4)	110/1234 (8.9)	2.70 (1.30–5.61)	0.008
Ex-smokers	4 (8.2)	83/1234 (6.7)	1.43 (0.50–4.13)	0.505
Non smokers	35 (71.4)	1041/1234 (84.4)	1	
Cooking fuel				
Biomass	1/43 (2.3)	98/1209 (8.1)	0.32 (0.04–2.46)	0.276
Mixed	25/43 (58.1)	571/1209 (47.2)	1.39 (0.74–2.60)	0.303
Clean	17/43 (39.5)	540/1209 (44.7)	1	
Past history of TB				
No	48 (98)	1218 (98.4)	1	
Yes	1 (2)	20 (1.6)	1.27 (0.17–9.65)	0.818
Past history of pneumonia				
No	47 (95.9)	1203 (97.3)	1	
Yes	2 (4.1)	34 (2.7)	1.51 (0.35–6.45)	0.582
HIV infection				
No	33 (67.3)	972 (78.5)	1	
Yes	1 (2)	10 (0.8)	2.95 (0.37–23.69)	0.310
Don’t know	15 (30.6)	256 (20.7)	1.73 (0.92–3.23)	0.087
Chronic cough				
No	48 (98)	1214 (98.1)	1	
Yes	1 (2)	24 (1.9)	1.05 (0.14–7.95)	>0.999
Chronic expectoration				
No	48 (98)	1229 (99.3)	1	
Yes	1 (2)	9 (0.7)	2.85 (0.35–22.91)	0.323
Chronic dyspnoea 1				
No	48 (98)	1181 (95.4)	1	
Yes	1 (2)	57 (4.6)	0.43 (0.06–3.18)	0.722
Lifetime wheezing				
No	42 (85.7)	1156/1234 (93.7)	1	
Yes	7 (14.3)	78/1234 (6.3)	2.47 (1.08–5.68)	0.038
BMI, kg, m(SD)	25.8 (4.4)	26.5 (5.45)	0.98 (0.92–1.03)	0.410

*m* mean, *SD* standard deviation, *TB* tuberculosis, *BMI* body mass index, *AFL* air flow limitation, *LLN* lower limit of normal

**Table 3:** Multivariable analysis of factors associated to air flow limitation and chronic obstructive lung disease

Factors	AFL–LLN		COPD–LLN	
	Adjusted OR (95 % CI)	p value	Adjusted OR (95 % CI)	p value
Sex				
Female	1		1	
Male	1.93 (1.00–3.73)	0.049	2.42 (1.12–5.20)	0.024
Age group, years				
19–39	1		1	
40–59	1.99 (1.04–3.81)	0.037	1.33 (0.57–3.09)	0.504
≥60	2.16 (0.79–5.92)	0.134	2.52 (0.82–7.73)	0.106
Tobacco smoking				
Non smokers	1		/	/
Current smoker	1.86 (0.84–4.07)	0.124	/	/
Ex-smokers	0.81(0.26–2.52)	0.713	/	/
HIV infection				
No	1		/	/
Yes	3.94 (0.47–33.21)	0.208	/	/
Don’t know	1.62 (0.85–3.06)	0.140	/	/
Lifetime wheezing				
No	1		1	
Yes	2.65 (1.13–6.20)	0.024	2.88 (1.06–7.81)	0.037

*TB* tuberculosis, *HIV* human immunodeficiency virus, *AFL* air flow limitation, *COPD* chronic obstructive pulmonary disease, *LLN* lower limit of normal, *OR* odd’s ration, *CI* confidence interval

Quality control was done by regularly supervising investigators and technicians who performed spirometry. Spirometry curves were reviewed weekly by one of chest physician who participated in the study (EWPY and ADB) and feedback was made to technicians.

### Determinants of COPD

Twenty-one (67.7 %) participants with COPD–LLN and 587 (47.1 %) participants without COPD–LLN were men (p = 0.023). The prevalence of lifetime wheezing was 16.1 % among those with COPD–LLN and 6.4 % among those without (p = 0.033), Table 4. No significant association was found between pulmonary tuberculosis, HIV status, BMI and COPD–LLN (Table 4). In multivariable analysis, male gender (AOR 2.42; 95 % CI 1.12–5.20) and lifetime wheezing (AOR 2.88; 95 % CI 1.06–7.81) were the determinants of COPD–LLN (Table 3).

**Table 4:** Factors associated to chronic obstructive pulmonary disease (COPD–LLN) in univariable analysis

Characteristics	COPD n = 31 (%)	No COPD n = 1245 (%)	OR (95 % CI)	p value
Sex				
Male	21 (67.7)	587 (47.1)	2.35 (1.10–5.04)	0.023
Female	10 (32.3)	658 (52.9)	1	
Age, years, m (SD)	38.5 (14.1)	34.2 (12.7)	1.02 (1.00–1.05)	0.071
Age group				
19–39	19 (61.3)	887 (71.2)	1	
40–60	8 (25.8)	292 (23.5)	1.28 (0.55–2.95)	0.564
≥60	4 (12.9)	66 (5.3)	2.83 (0.94–8.56)	0.066
Level of education				
≤Secondary	21/30 (70)	825/1244 (66.3)	1.19 (0.54–2.61)	0.673
Higher education	9/30 (30)	419/1244 (33.7)	1	
Subject/chamber				
≤2	16 (61.6)	695/1243 (55.9)	1	0.634
>2	15 (48.4)	548/1243 (44.1)	0.84 (0.41–1.72)	
Tobacco smoking				
Current smoker	4 (12.9)	113/1241 (9.1)	1.55 (0.53–4.52)	0.432
Ex-smokers	3 (9.7)	84/1241 (6.8)	1.55 (0.46–5.27)	0.479
Non smokers	24 (77.4)	1044/1241 (84.1)	1	
Cooking fuel				
Clean	9/27 (33.3)	544/1214 (44.8)	1	
Biomass	1 (3.7)	97 (8)	0.62 (0.08–4.97)	0.655
Mixed	17 (63)	573 (47.2)	1.79 (0.79–4.06)	0.161
Past history of TB				
No	30 (96.8)	1226/1245 (98.5)	1	
Yes	1 (3.2)	19/1245 (1.5)	2.15 (0.28–16.60)	0.391
Past history of pneumonia				
No	30 (96.8)	1209/1244 (97.2)	1	
Yes	1 (3.2)	35/1244 (2.8)	1.15 (0.15–8.68)	0.891
HIV infection				
No	20 (64.5)	977/1245 (78.5)	1	
Yes	1 (3.2)	10/1245 (0.8)	4.86 (0.60–40.00)	0.139
Don’t know	10 (32.3)	258/1245 (20.7)	1.89 (0.88–4.10)	0.105
Chronic cough/expectoration				
No	30 (96.8)	1219/1245 (97.9)	1	
Yes	1 (3.2)	26/1245 (2.1)	1.56 (0.21–11.89)	0.489
Chronic dyspnea				
No	30 (96.8)	1188/1245 (95.4)	1	
Yes	1 (3.2)	57/1245 (4.6)	0.70 (0.09–5.19)	>0.999
Lifetime wheezing				
No	26 (83.9)	1161/1241 (93.6)	1	
Yes	5 (16.1)	80 (6.4)	2.79 (1.04–7.46)	0.033
BMI, kg, m(SD)	27.2 (4.6)	26.4 (5.5)	1.02 (0.96–1.09)	0.445

*OR* odd’s ration, *M* mean, *SD* standard deviation, *TB* tuberculosis, *BMI* body mass index, *COPD* chronic obstructive pulmonary disease, *LLN* lower limit of normal

## Discussion

In this study conducted in a major city in sub-Saharan Africa using internationally accepted criteria to define AFL and COPD, the main findings were the following: 1) the prevalence of airway obstruction was 3.8 and 0.7 % using respectively the LLN and fixed ratio of 0.70 for FEV1/FVC to define airway obstruction; 2) the prevalence of COPD was 2.4 and 0.5 % using respectively the LLN and fixed ratio to define COPD; 3) determinants of AFL–LLN were male sex, age, and wheezing, while 4) male sex and wheezing were the determinants of COPD–LLN.

The few available studies on OLD in urban Africa using a pre-bronchodilator FEV1/FVC <0.70 to define airway obstruction [23, 24] include a study from Nigeria in 2002 and a study from South Africa in 2009 in which the prevalences of airway obstruction were 9.3 and 26 % respectively. In the USA, the prevalence of bronchial obstruction based on a fixed pre-bronchodilator ratio has been reported to be about 20.9 % [25]. These rates were higher than those found in our study. Globally, the prevalence of airway obstruction based on pre-bronchodilator’s LLN ranges between 9 and 24 % in the 40 year and above age group [24]. The rate of airway obstruction in our study based on pre-bronchodilator’s LLN, both in the total population and in those aged 40 years and above, were at the lower tail of reported figures from around the world.

The prevalence of COPD based on a fixed post-bronchodilator ratio was 0.5 % in our population, which is lower than the 11–25 % reported rate from other regions around the world [22, 24, 26]. Based on a more appropriate definition for our young population (i.e. post-bronchodilator LLN) to diagnose COPD, the prevalence was 2.1 % in the general population and 3.2 % among those aged 40 years and above [27, 28]. This was lower than the 7–20 % reported elsewhere [24]. However, it was close to the 4.5 % reported by Musafari et al. in Rwanda based on the same definition [22].

The overall low prevalence of airway obstruction in our study regardless of diagnostic criteria is at least in part explained by the low prevalence of smoking in our population. In this study, the prevalence of current and former smoking was 16.2 % and only 15.6 % of smokers and ex-smokers had a cumulative quantity of smoked of 20 pack-years or more. This is at variance with the very high prevalence of smoking in regions with high prevalence of COPD [24, 25]. The low prevalence of COPD in our study could also be explained by the young age of our sample. This young age however is in keeping with the age structure of the Cameroon population [14]. COPD is more frequent in men than in women in our study in accordance with studies from developed countries [29]. This sex differential was however not found in a recent systematic review conducted by Adeloye et al., which included studies from sub-Saharan Africa [30]. Other well-known risk factors for COPD such as pulmonary tuberculosis, HIV infection, exposure to biomass were not associated with LLN–COPD in our study, likely reflecting the low statistical power due to the low prevalence of those characteristics in the general population.

The potential limitations of this study include the small number of participants’ age 40 years and above, and the inclusion of participants exclusively from urban areas. However, considering that non-tobacco related determinants of COPD can operate at any age, the inclusion in prevalence studies of COPD of a much broader age range is justified. Exposure to biomass is usually more important in rural than urban areas, and it is possible that including rural participants could modify some of our findings. van Gemert et al. have recently found the COPD prevalence to be 16.2 % in rural Uganda region with high level of biomass utilisation [31]. Our study also has major strengths. It is the first study in Cameroon and the entire central Africa region to use spirometry to assess the population burden of COPD. It is therefore a useful addition to existing studies on this topic from Africa [6, 30].

## Conclusions

Airway obstruction was relatively low in this central African population with low smoking habits, with substantial evidence that the prevalence increased with advanced age. It is important to sensitize the population and decision maker on this likely growing health treats. Larger studies comprising rural participants are needed to refine the findings from the current study.

## Abbreviations

AFL: air flow limitation
ATS/ERS: American Thoracic Society/European Respiratory Society
COPD: chronic obstructive pulmonary disease
EA: enumeration areas
FEV1: forced expiratory volume in 1 s
FVC: forced vital capacity
GOLD: global initiative for chronic obstructive lung disease
LLN: lower limit of normal
OLD: obstructive lung disease

## References

[1] World Health Organisation: Burden of COPD (2015), http://www.who.int/respiratory/copd/burden/en/. Accessed 22 Feb 2015.

[2] van Schayck CP, Chavannes NH (2003). Detection of asthma and chronic obstructive pulmonary disease in primary care. Eur Respir J Suppl.

[3] Gupta RP, Perez-Padilla R, Marks G, Vollmer W, Menezes A, Burney P (2014). Summarising published results from spirometric surveys of COPD: the problem of inconsistent definitions. Int J Tuberc Lung Dis.

[4] Johnson P, Balakrishnan K, Ramaswamy P, Ghosh S, Sadhasivam M, Abirami O (2011). Prevalence of chronic obstructive pulmonary disease in rural women of Tamilnadu: implications for refining disease burden assessments attributable to household biomass combustion. Glob Health Action.

[5] Gingo MR, Balasubramani GK, Rice TB, Kingsley L, Kleerup EC, Detels R (2014). Pulmonary symptoms and diagnoses are associated with hiv in the MACS and WHIS cohorts. BMC Pulm. Med..

[6] Finney LJ, Feary JR, Leonardi-Bee J, Gordon SB, Mortimer K (2013). Chronic obstructive pulmonary disease in sub-Saharan Africa: a systematic review. Int J Tuberc Lung Dis.

[7] World Health Organisation: Tobacco control–overview (2014), http://www.afro.who.int/en/clusters-a-programmes/hpr/health-risk-factors/tobacco/overview.html. Accessed 17 Oct 2014.

[8] Pai M, Lee C-H, Lee M-C, Lin H-H, Shu C-C, Wang J-Y (2012). Pulmonary tuberculosis and delay in anti-tuberculous treatment are important risk factors for chronic obstructive pulmonary disease. PLoS One.

[9] Arjomandi M, Faner R, Gonzalez N, Cruz T, Kalko SG (2014). Systemic inflammatory response to smoking in chronic obstructive pulmonary disease: evidence of a gender effect. PLoS One.

[10] World Health Organisation: The 10 leading causes of death by country income group (2012) http://www.who.int/mediacentre/factsheets/fs310/en/index1.html. Accessed 12 Dec 2014.

[11] Lopez AD, Mathers CD, Ezzati M, Jamison DT, Murray CJ (2006). Global and regional burden of disease and risk factors, 2001: systematic analysis of population health data. Lancet.

[12] Global initiative for chronic obstructive lung disease (GOLD): Global strategy for the diagnosis, management and prevention of COPD (2013), http://www.goldcopd.org. Accessed 10 Oct 2014.

[13] Pellegrino R, Viegi G, Brusasco V, Crapo RO, Burgos F, Casaburi R (2005). Interpretative strategies for lung function tests. Eur Respir J.

[14] Bureau Central des Recensements et des Etudes de Population: Rapport national sur l’etat de la population (2011), http://www.bucrep. Accessed 21 Sep 2014.

[15] Organisation Mondiale de la Santé. Les jeunes et la santé: Défi pour la société (1986), http://whqlibdoc.who.int/trs/WHO_TRS_731_fre.pdf. Accessed 15 Jan 2015.

[16] Buist AS, Vollmer WM, Sullivan SD, Weiss KB, Lee TA, Menezes AM (2005). The burden of obstructive lung disease initiative (bold): rationale and design. COPD.

[17] Ferris BG (1978). Epidemiology standardization project (American Thoracic Society). Am Rev Respir Dis.

[18] Miller MR, Hankinson J, Brusasco V, Burgos F, Casaburi R, Coates A (2005). Standardisation of spirometry. Eur Respir J.

[19] Brusasco VCRVG (2005). Standardisation des explorations fonctionnelles respiratoires. Eur Respir J.

[20] Musafiri S, van Meerbeeck JP, Musango L, Derom E, Brusselle G, Joos G (2013). Spirometric reference values for an East-African population. Respiration..

[21] Global Initiative for Chronic Obstructive Lung Disease (GOLD): Global strategy for the diagnosis, management, and prevention of COPD. Executive summary (2007), http//www.goldcopd.org/uploads/users/files/GOLDReport07_0108.pdf. Updated 2007. 10.1081/COPD-120030163 16997745

[22] Musafiri S, van Meerbeeck J, Musango L, Brusselle G, Joos G, Seminega B (2011). Prevalence of atopy, asthma and COPD in an urban and a rural area of an African country. Respir Med.

[23] Gathuru IM, Bunker CH, Ukoli FA, Egbagbe EE. Differences in rates of obstructive lung disease between africans and african americans. Ethn Dis. 2002;12:S3-107-113. 12477165

[24] Vollmer WM, Gislason T, Burney P, Enright PL, Gulsvik A, Kocabas A (2009). Comparison of spirometry criteria for the diagnosis of copd: results from the bold study. Eur Respir J.

[25] Tilert T, Dillon C, Paulose-Ram R, Hnizdo E, Doney B (2013). Estimating the U.S. prevalence of chronic obstructive pulmonary disease using pre- and post-bronchodilator spirometry: the National Health And Nutrition Examination Survey (NHANES) 2007-2010. Respir Res.

[26] Ching SM, Pang YK, Price D, Cheong AT, Lee PY, Irmi I, Faezah H, Ruhaini I, Chia YC (2014). Detection of airflow limitation using a handheld spirometer in a primary care setting. Respirology.

[27] Cerveri I, Corsico AG, Accordini S, Niniano R, Ansaldo E, Anto JM (2008). Underestimation of airflow obstruction among young adults using FEV1/FVC <70 % as a fixed cut-off: a longitudinal evaluation of clinical and functional outcomes. Thorax.

[28] Swanney MP, Ruppel G, Enright PL, Pedersen OF, Crapo RO, Miller MR (2008). Using the lower limit of normal for the FEV1/FVC ratio reduces the misclassification of airway obstruction. Thorax.

[29] Buist AS, Vollmer WM, McBurnie MA (2008). Worldwide burden of COPD in high- and low-income countries. Part 1. The burden of obstructive lung disease (BOLD) initiative. Int J Tuberc Lung Dis.

[30] Adeloye D, Basquill C, Papana A, Chan KY, Rudan I, Campbell H (2015). An estimate of the prevalence of COPD in Africa: a systematic analysis. COPD.

[31] van Gemert F, Kirenga B, Chavannes N, Kamya M, Luzige S, Musinguzi P (2015). Prevalence of chronic obstructive pulmonary disease and associated risk factors in Uganda (FRESH AIR Uganda): a prospective cross-sectional observational study. Lancet Glob Health..

